# Elucidating breed-specific variants of native pigs in Korea: insights into pig breeds’ genomic characteristics

**DOI:** 10.1080/19768354.2022.2141316

**Published:** 2022-11-08

**Authors:** Young-Sup Lee, Seungwoo Son, Hak-Kyo Lee, Ra Ham Lee, Donghyun Shin

**Affiliations:** a Department of Animal Biotechnology, Jeonbuk National University, Jeonju, Republic of Korea; b Department of Agricultural Convergence Technology, Jeonbuk National University, Jeonju, Republic of Korea

**Keywords:** Breed-specific SNPs, breed-informative, genomic characteristics, native pigs, whole genome sequencing

## Abstract

Although conserving native pig breeds is important in Korea, research on the genomic aspects to identify breed-specific variations in native pig breeds is uncommon. Single nucleotide polymorphisms (SNPs) can be a powerful source for identifying breed-specific variants. We used whole genome sequencing data, including Jeju Native Pig (JNP), Korean Native Pig (KNP), Korean Wild Boar (KWB), and other western commercial pig breeds to determine native pig breed-specific SNPs. Furthermore, the goal was not only to determine the genomic specificity of native pig breeds but also to identify SNPs that carry breed-specific information (breed-informative SNPs) that can be related to breed characteristics. The representative characteristics of native pigs are their unique meat quality and disease resistance. We surveyed the gene ontology (GO) of native pigs with breed-specific SNPs. Examining the genes associated with GO may contribute to revealing the reasons for the unique characteristics of native pig breeds. The enriched GOs terms were neuron projection development, cell surface receptor signaling pathway, ion homeostasis in JNP, cell adhesion and wound healing in KNP, and DNA repair and reproduction in KWB. We expect that this study of breed-specific SNPs will enable us to gain a deeper understanding of native pigs in Korea.

## Introduction

Pigs were first domesticated in the Near East around 8500 BC and were brought into Europe by agriculturalists (Larson et al. 2007). Evidence supports the independent domestication of pigs in Asia and Europe from the wild boar subspecies (Giuffra et al. 2000). The answer to which pig domestication was independent or connected by diffusion from a single origin remains to be established, although recent research based on complete mitochondrial genomes of East Asian pigs suggests that wild boar in China may have been domesticated independently in the Mekong and in the middle to downstream Yangtze regions (Larson et al. 2010). Recently, western pig breeds have been imported into the Korean peninsula and crossed with native pigs in Korea to improve their growth and carcass-related traits, which has led to a dramatic drop in the native pig population. Although commercial pig breeds are superior in terms of growth and feed efficiency traits, native pigs in Korea are known for their better meat quality (high redness and intramuscular fat) and greater ability to thrive under low management conditions (Yeo et al. 2000, Kim et al. 2005).

Single nucleotide polymorphisms (SNPs) have been used to describe genetic variability and identify phenotype-associated candidate genes. Furthermore, SNPs can capture breed differences and be used to determine the breed of each pig. Informative SNPs have been used for comparative selection signature analyses, parentage assessment, and breed assignment of individual animals, as well as for several other applications (Kuehn et al. 2011, Asaf et al. 2014). The genomic diversity of specific breeds with specific phenotypic and thus genotypic characteristics can be uncovered in some ways (Czech et al. 2018). Determining breed-specific variants is one method for uncovering genetic characteristics. Breed-specific variants, including SNPs and nonsynonymous SNPs (nsSNPs), can not only differentiate the target breeds from other breeds but also reveal their characteristics. More specifically, breed-specific nsSNPs alter the amino acid sequence and protein function and can play a role in delineating breed characteristics (Lee, Won et al. 2020).

The native pigs in Korea (JNP: Jeju native black pig, KNP: Korean native pig, KWB: wild boar in Korea) differ from the Western commercial breeds in appearance; their fur is black, the ears are folded forward, and are resistant to disease. They have a unique meat quality, which Koreans prefer. Specifically, JNP, a native pig breed in Korea, has survived for a long time without mixing with other pig breeds, in an independent environment away from inland. Since the 1960s, the number of JNPs has sharply declined, putting them on the verge of extinction. Since 1986, the pure line breeding project has been initiated, and ∼ 260 black pigs have been preserved and managed. KWB has a maximum body length of 180 cm and weight ranging 50–300 kg. Their legs are short and slender, and they run fast. Body color varies widely from dark brown to light brown. To reveal the unique characteristics of Korea's native pig breeds, we investigated the specific variants of the three breeds to determine the genomic cause of their characteristics. The breed-specific variants can be breed-informative. Kumar et al. attempted to determine cow breed-specific SNPs in India that are associated with the breeds’ characteristics, such as milk production, heat tolerance, disease resistance, and fertility (Kumar et al. 2021). Ramos et al. developed breed-specific SNPs in pig breeds, presenting breed-specific markers for breed assignment and traceability (Ramos et al. 2011).

Purebred pigs are commercially important, and many breeders have requested them in their cross-breeding programs. Cross-breeding helps breeders discover new breeds with desirable traits, such as disease resistance and heat tolerance (Pasupa et al. 2020). When crossing native pigs in Korea with western commercial pig breeds, we aimed to determine the breed-specific variants of native pigs in Korea, which could help improve the conservation of the genetic resources of native breeds. We used whole genome sequencing data from native pig breeds, western commercial pig breeds, and variant information. The breed-specific SNPs and breed-specific nonsynonymous SNPs (nsSNPs) of native pig breeds were identified, and the characteristics of the SNPs were surveyed using gene ontology (GO) analysis.

## Materials and methods

### Whole genome sequencing

The pig fastq sequence data—Berkshire (BKS) 10, Duroc (DUR) 20, Jeju Native Pig (JNP) 20, Korean native pig (KNP) 6, Korean wild boar (KWB) 10, landrace (LDR) 13, Yorkshire (YKS) 15, and Yucatan Miniature Pig (YMP) 12—were checked using the FastQC software. Potential adapter sequences were removed using Trimmomatic-0.32 (Bolger et al. 2014), followed by mapping paired-end sequence reads to the pig reference genome (Sscrofa 11.1) obtained from Ensembl. Picard tools (http://broadinstitute.github.io/picard/), SAMtools (Li and Durbin 2009), and the Genome Analysis Toolkit (GATK) (McKenna et al. 2010) were used for downstream processing and variant calling. Using ‘CreateSequenceDictionary’ and ‘MarkDuplicates’ Picard command-line tools, we read reference FASTA sequences to generate bam files with only a sequence dictionary and filter potential PCR duplicates. The index files for the reference and BAM files were created using SAMtools. Using the GATK ‘Realigner-TargetCreator’ and ‘IndelRealigner’ arguments, local realignment of the sequence reads was performed to remove small insertions and deletions. Additionally, the base quality score was recalibrated to obtain accurate quality scores and correct for the variation in quality with the machine cycle and sequence context. To call variants, GATK ‘UnifiedGenotyper’ and ‘SelectVariants’ arguments were used with the following standards: 1) Phred-scaled quality score of less than 30, 2) read depth less than 5, 3) MQ0 (total count of mapping quality zero reads across all samples) > 4, and 4) a Phred-scaled *P*-value using Fisher's exact test of more than 200 were filtered out to reduce false-positive calls due to strand bias.

The SNPs dataset (37,823,235) was imputed using Beagle 5.3 (Browning and Browning 2007), and QC (Quality Check) was performed with minor allele frequency (MAF < 0.05) and Hardy-Weinberg Equilibrium (HWE *p*-value < 1.0E-06), leaving 16,868,444 SNPs. Figures were drawn using the data after QC. Nevertheless, to determine the breed-specific SNPs, untreated data was used before QC. The SNPs were annotated using the SNP annotation tool SnpEff (version 4.1) (Cingolani et al. 2012).

### Examining the population structure of native pig breeds in Korea

To verify the purity of each pig breed, we analyzed its genomic structures. The selection of JNP-, KNP-, and KWB-breed-specific SNPs should be based on the purity of each breed. Admixture analysis to check the purity of the pig breeds was performed using fastStructure, based on a variational Bayesian framework, and a genetic cluster of size 8 (Raj et al. 2014) was estimated. Breeds were classified as pure for the most part, but 5 JNP individuals were mixed with the DUR breed. Therefore, we excluded five individuals from the subsequent analysis.

As a dimension reduction method, principal component analysis (PCA) combines multiple features into lower-dimensional features, that can explain most of the variance in a large dataset (Lee et al. 2021). PCA was conducted using genome-wide complex trait analysis (GCTA) to obtain eigenvalues and eigenvectors (Yang et al. 2011) and to assess the explanatory power of each breed-specific SNPs, which revealed that breed-specific SNPs can distinguish them from other breeds. The neighbor-joining tree (NJ tree) was constructed using a distance matrix calculated using VCF2Dis (https://github.com/BGI-shenzhen/VCF2Dis) and MEGA6 (https://megasoftware.net/) (Tamura et al. 2013). Treemix v1.13 was used to identify historical relationships among pig populations (Fitak 2021)

### Identifying breed-specific SNPs

(1) $$breed-specificvariant:\frac{A^{\prime }\;allele\;count}{total\;allele\;count}inobjectpopulation>0.9\&\frac{A\;allele\;count}{total\;allele\;count}inreferencepopulation>0.9$$

where A is reference allele or alternative allele and total allele count was defined to each object population and reference population.

A comparison of the number of reference and alternative alleles was conducted to determine the breed-specific variants of native Korean pigs (JNP, KNP, and KWB). In three cases, the allele count was the criterion used to select breed-specific SNPs. For instance, in KWB pig breeds, if KWB possessed more than 90% alternative alleles in KWB and more than 90% of the reference allele in other pigs and vice versa, the SNPs were selected as KWB-specific SNPs (Equation 1). JNP and KNP had the same conditions as those of KWB. However, for JNP and KNP, we excluded KWB from the analysis because the alleles of the native pigs in Korea (JNP and KNP) can overlap with the recent common ancestors of KWB.

### Breed-specific nonsynonymous SNPs (nsSNPs)

In JNP, breed-specific SNPs and encompassing genes were used for further analyses. Enormous SNPs were not detected as breed-specific SNPs in comparison with other native pigs. However, numerous breed-specific SNPs have been detected in the KNP and KWB. Thus, breed-specific SNPs that overlapped with nonsynonymous SNPs (nsSNPs) were used for the subsequent analysis. nsSNPs are an important type of SNPs that alter amino acid sequences and potentially affect protein structure and function (Krawczak et al. 2000, Wu and Jiang 2013). These breed-specific nsSNPs are likely to link pig phenotypes to genomic variations. To predict whether SNPs were nonsynonymous, we used the SnpEff program with the reference genome version Sscrofa 11.1. SnpEff is a variant annotation and effect prediction tool that is used to identify differences like amino acid changes (Cingolani, Platts, Wang, Coon, Nguyen, Wang, Land, Lu and Ruden 2012).

### GO analysis

We analyzed the GO using breed-specific SNPs. For JNP, genes encompassing breed-specific SNPs were used for GO analysis. Genes encompassing breed-specific nsSNPs were selected for KNP and KWB. The gene catalogue was retrieved from the Ensembl DB (http://www.ensembl.org) and Database for Annotation, Visualization, and Integrated Discovery (DAVID 2021). A list of gene identifiers was uploaded to summarize the functional annotations associated with groups or each individual gene, and each biological process (BP)-related GO term was based on the number of genes and *p*-values (Huang et al. 2007).

## Results

### SNP data description

**Figure 1.**
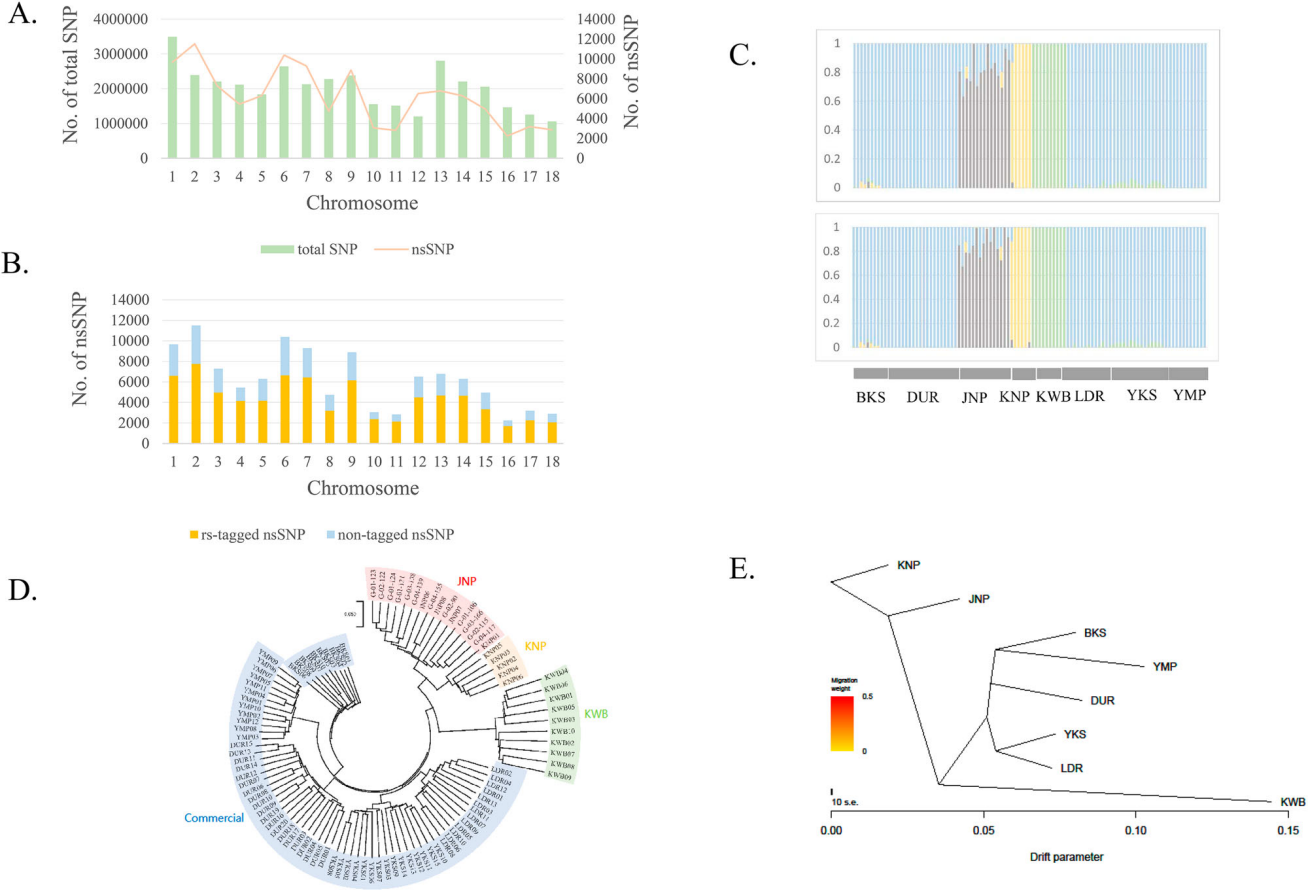
(A) No. of total SNPs (orange bar) and nsSNPs (blue line) across autosomes in whole genome sequencing data of pig breeds. (B) The rs-tagged nsSNPs (orange bar) and non-tagged nsSNPs (blue bar) across autosomes. (C) The structure analysis with the parameter K = 4 were performed with total SNPs after quality control (upper panel) and breed-specific SNPs (lower panel). There were no differences between the two cases and it revealed that breed-specific SNPs set could be the representatives of total SNPs that could show breed-specificity. (D) The NJ tree of pig breeds are Jeju Native Pig (JNP), Korean Native Pig (KNP), Korean Wild Boar (KWB), Berkshire (BKS), Yorkshire (YKS), Duroc (DUR), Landrace (LDR), Yucatan Miniature Pig (YMP). It showed the distinctness of each breed. (E) The plot was drawn using Treemix program and could show the historical relationships among the populations. It showed that JNP, KNP and KWB were separate with western commercial pig breeds.

The number of SNPs per autosome (chromosome 1–18) ranged from 1,056,274–3,493,244. The number of nsSNPs ranged from 2,868–11,507, while the number of SNPs and nsSNPs per autosome tended to co-vary (Figure 1A). The average distance between adjacent SNPs per autosome (± standard deviation [SD]) ranged from 100 (± 181) to 186 (± 291). The number of rs-tagged nsSNPs ranged from 1,687–7,754, and the number of novel nsSNPs ranged from 551–3,753 (Figure 1B).

### Population stratification

Structural analysis using total SNPs and setting the parameters K to 6, 7, and 8 revealed that some JNPs were mixed with DUR in five JNP individuals; thus, these five JNP pigs were eliminated from the analysis. After elimination, the purity of each breed was verified by structural analysis. Analyses with the parameter K = 4, using total SNPs after QC (upper panel) and breed-specific SNPs (lower panel), were performed to check the representativeness of breed-specific SNPs (Figure 1C). In the NJ tree, each native pig breed (JNP, KNP, and KWB) was distinct from other commercial pig breeds (Figure 1D). For Treemix, the results were congruent with those of the NJ tree (Figure 1E).

### Breed-specific SNPs

**Figure 2.**
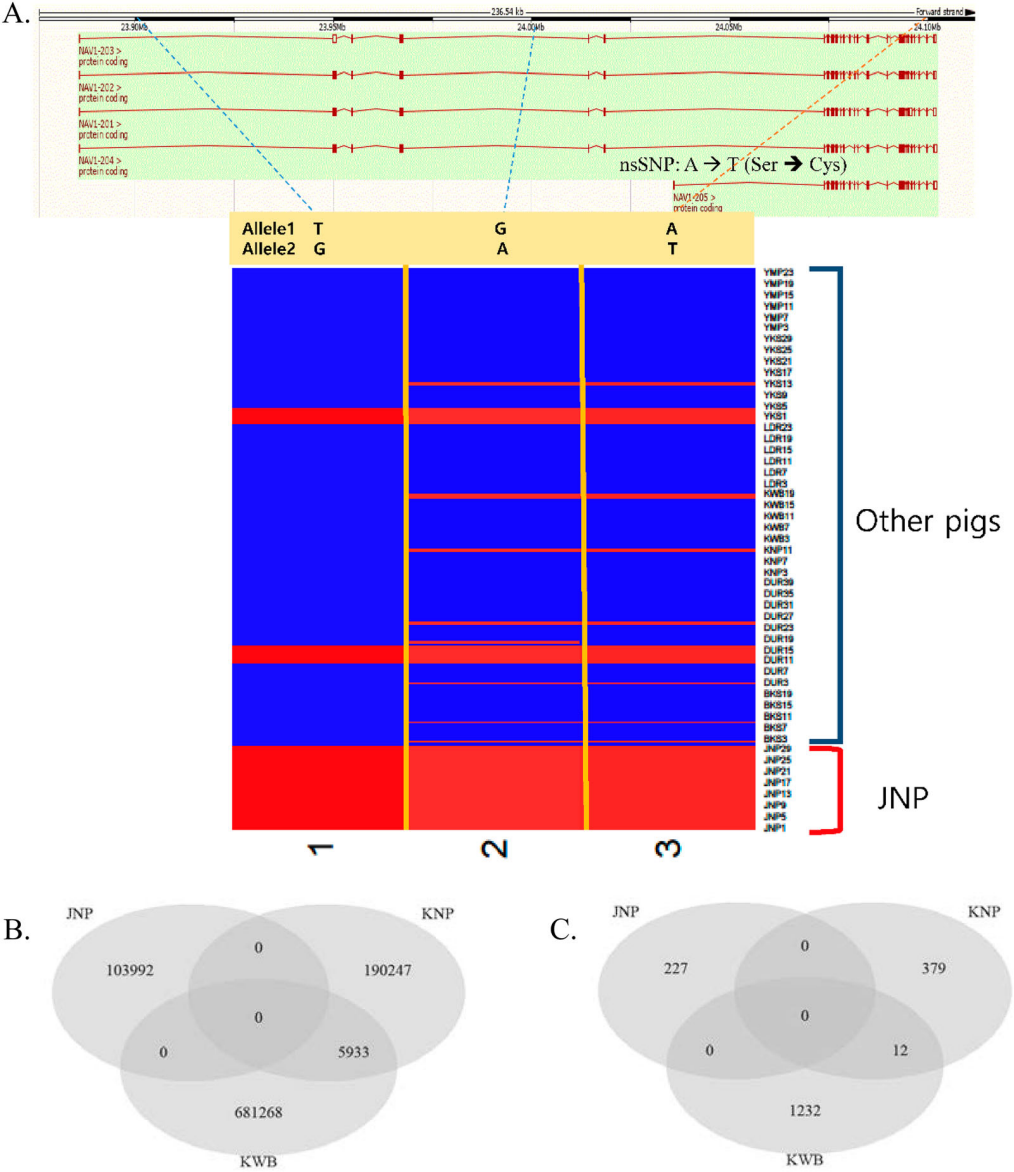
(A) The three JNP breed-specific SNPs in Neuron Navigator 1 (NAV1; top) gene including nsSNP (A → T, Ser → Cys). The NAV1 gene were illustrated because of one of the top genes as JNP-specific SNPs (In all three SNPs, JNP individuals’ alleles were unity). (B) Venn diagram of the total number of breed-specific SNPs in JNP, KNP and KWB. There were no overlaps between JNP and KNP, KWB. (C) Venn diagram of the total number of breed-specific nonsynonymous SNPs (nsSNPs) in JNP, KNP and KWB.

**Figure 3.**
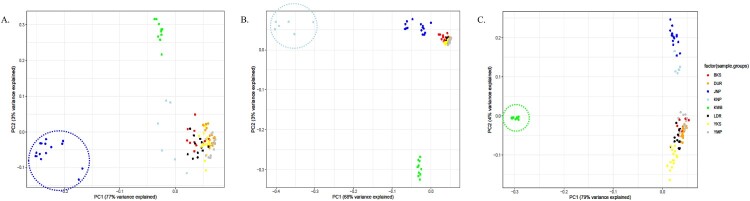
The principal component analysis (PCA) plot using JNP (A), KNP (B) and KWB (C) -specific SNPs. These PCA plot show that the breed-specific SNPs play a characteristic role in defining the breeds. The individuals in JNP, KNP and KWB were color-circled, respectively. The principal component 1 (PC1) was the determining factor in each PCA plot.

To identify the genomic characteristics of the native pig breeds in Korea (JNP, KNP, and KWB), breed-specific SNPs and nsSNPs were surveyed. Most breed-specific SNPs in JNP, KNP, and KWB were from alternative alleles, indicating the divergence of native pig breeds from wild boar ancestors. The three SNPs encompassed in neuron navigator 1 (NAV1) were JNP-specific SNPs that showed the unity of JNP alleles (Figure 2A). The Venn diagram illustrating breed-specific SNPs and nsSNPs showed that ∼ 0.3–2% of the total SNPs could be classified as breed-specific. The number of breed-specific SNPs in JNP, KNP, and KWB was 103,992, 196,180, and 687,201, respectively. The number of breed-specific nsSNPs was 227, 391, and 1,244, respectively. In particular, breed-specific SNPs and nsSNPs of JNP did not overlap with those of KNP and KWB (Figure 2B and C). It is likely that the JNP diverged well from the wild boars in Korea. PCA analysis showed that the breed-specific SNPs in JNP (Figure 3A) and the breed-specific nsSNPs in KNP and KWB (Figure 3B and C) were distinct from other pig breeds.

### GO analysis for native pig breeds in Korea

Gene ontology analysis of the genes encompassing JNP-specific SNPs revealed that neuron projection development (GO:0031175), cell surface receptor signaling pathway (GO:0007166), and ion homeostasis (GO:0050801) were the most frequent GO terms (Table 1). In the KNP-specific nsSNPs, cell adhesion (GO:0007155) and wound healing (GO:0042060) were the most frequent GO terms (Table 2). The JNP and KNP unique pork quality can originate from genes that were affected by breed-specific variants. In KWB, the enriched GOs pertained to KWB-specific nsSNPs and the genes for DNA repair (GO:0006281) and reproduction (GO:0000003) (Table 3).

**Table 1.** The table of Gene ontology (GO) of the genes encompassing JNP-specific SNPs. The frequent terms were cell surface receptor signaling pathway, neuron projection development, and ion homeostasis.

Term	Count	*P*-Value	Fold Enrichment	Genes
GO:0007166∼cell surface receptor signaling pathway	127	2.26E-04	1.4	HHIP, CTNND1, HHAT, RASSF2, KDR, ENPP1, PDK2, MED1, SEMA6A, FBXW7, IL1R2, CD180, MUL1, PRKCA, HNF1B, RUNX2, DKK3, DEPDC1B, SFRP2, CCNY, SMO, ADGRB2, ROR1, IL6ST, IFNAR1, BLK, GIPR, NPR2, CTR9, PRDM14, TGFA, NREP, DEDD2, CD79B, CD79A, CALCR, KLRK1, ADGRG6, FGF20, EIF4EBP2, ANKRD10, LPXN, PLCG1, PLXNA4, WNT3, FAM83B, PRNP, TGFB2, SMAD3, FZD4, SMAD6, PTK2, BMP6, BMP5, IL22RA1, ZC3HC1, GH1, CXCL12, FGF18, ITGA11, AAK1, GRB2, OSTN, GLP1R, ITGB5, DDX47, PTPRO, CELF4, SPPL3, SH3RF1, ROBO1, GLI2, GHR, LRRTM4, LRRTM1, BOC, IQUB, DACT2, JAK3, PHACTR4, ANXA4, PDE4D, GLP2R, GAB1, RGMB, LAT2, F7, ADGRF1, ADGRF4, KIT, ADGRF5, GFRAL, ITGA8, CLDN18, PPARG, IRF5, ITGA5, GAS6, ZMYND11, TLR2, LEPROT, CCL11, PRKAA2, SGMS1, HPGD, PRICKLE1, TSPAN33, PROKR2, CUX2, STAT6, XDH, IL12RB2, RYK, STAT3, SEMA4F, BAIAP2, SULF1, APC, LEP, OTULIN, KCP, F2RL1, SPRY1, GHRL, ADGRL4
GO:0031175∼neuron projection development	49	6.38E-04	1.6	ITSN2, TOP2B, PTPRO, STMN2, KNDC1, ROBO1, GLI2, SYNGAP1, ZFYVE27, CAPZB, KIF5C, DPYSL2, TCTN1, BOC, EFHD1, EPHB2, PRKG1, CAMK1D, SEMA6A, FBXW8, MAP1S, MUL1, UNC5D, KEL, AMIGO1, PLPPR4, PLPPR5, LPAR3, ADCY1, BLOC1S5, CUX2, PTPRZ1, ZSWIM6, WNT3, PLXNA4, PACSIN1, NTNG1, TGFB2, RYK, CCK, LAMB1, BRAF, SEMA4F, BAIAP2, BMP5, LRFN2, CXCL12, KIF26B, GHRL
GO:0050801∼ion homeostasis	45	7.88E-04	1.7	GLP1R, CAMK2D, ITPR3, SPPL3, ADRA1A, CLN6, SLC4A5, CACNA1G, SCGN, CALCR, RAB20, ATP6V0A2, CDH23, KDR, TAC4, ENPP1, CAPN3, PLCG1, CNNM2, PDK2, ATP6V0A1, SWAP70, TRPC1, PDE4D, ATP2B1, SYPL2, GPCPD1, CP, ESR1, BMP6, HCRTR2, SLC9A2, SLC4A7, SLC9A4, KEL, TRPV4, SLC9A9, AGTR1, F2RL1, GHRL, FAM20A, GPD1L, SLC26A4, ISCU, CFTR
GO:0044089∼positive regulation of cellular component biogenesis	32	8.78E-04	1.9	CCL11, CLSTN2, AMIGO1, PLPPR5, WASL, FNBP1L, SCIN, CUX2, LRRTM1, SYNDIG1, KDR, SNX9, ARFIP1, EPHB2, BCAS3, SMAD3, GSN, CNOT6L, SWAP70, BRAF, HNF1B, VPS37B, TERF1, BAIAP2, MPP7, CLIP1, PSMC3, CDC42EP4, F2RL1, GRB2, GHRL, EPHA1
GO:0006885∼regulation of pH	12	0.0012	3.1	SLC4A7, SLC9A2, SLC9A4, RAB20, SLC9A9, ATP6V0A2, SLC26A4, CLN6, CFTR, PDK2, SLC4A5, ATP6V0A1
GO:0051222∼positive regulation of protein transport	30	0.0012	1.9	BLK, SPPL3, WDR46, IL1RL1, RANBP3L, HHAT, MED1, BCAS3, TGFB2, SMAD3, FBXW7, ACTL6A, ACSL3, SREBF2, PTK2, GCK, BMP6, GLUD1, SFRP2, SMO, TRPV4, LEP, F2RL1, PPARG, GHRL, SAE1, GAS6, CFTR, TLR2
GO:0030030∼cell projection organization	69	0.0019	1.4	ITSN2, CEP126, TOP2B, SPEF2, PTPRO, STMN2, KNDC1, FNBP1L, ROBO1, GLI2, SYNGAP1, ZFYVE27, CAPZB, KIF5C, DPYSL2, IQUB, TCTN1, BOC, EFHD1, EPHB2, PRKG1, BCAS3, CAMK1D, SEMA6A, FBXW8, MAP1S, MUL1, EMP3, UNC5D, WDR35, VAV2, SFRP2, KEL, CDC42EP4, MAK, KIT, ITGA8, AMIGO1, PLPPR4, PLPPR5, LPAR3, ADCY1, RAP1GAP, BLOC1S5, CUX2, PTPRZ1, CDH23, ZSWIM6, WNT3, PLXNA4, PACSIN1, NTNG1, TGFB2, GSN, RYK, CCK, LAMB1, BRAF, SEMA4F, BAIAP2, BMP5, TSGA10, LRFN2, CXCL12, KIF26B, F2RL1, GHRL, CEP41

**Table 2.** The table of GO of the genes encompassing KNP-specific nsSNPs. The main terms were cell adhesion and wound healing.

Term	Count	*P*-Value	Fold Enrichment	Genes
GO:0007155∼cell adhesion	13	0.019	2.1	COL26A1, PRKDC, ITGB3, ZAN, IL27, GP1BA, LY9, SELP, ACAN, ISLR, PECAM1, CCR7, KIFAP3
GO:0022610∼biological adhesion	13	0.02	2.1	COL26A1, PRKDC, ITGB3, ZAN, IL27, GP1BA, LY9, SELP, ACAN, ISLR, PECAM1, CCR7, KIFAP3
GO:0042060∼wound healing	6	0.02	3.8	SELP, ITGB3, PECAM1, SDC1, GP1BA, F5
GO:0009988∼cell-cell recognition	3	0.024	12.2	CCT2, ZAN, CCR7
GO:0009611∼response to wounding	6	0.038	3.2	SELP, ITGB3, PECAM1, SDC1, GP1BA, F5
GO:0030534∼adult behavior	4	0.038	5.3	CSTB, SDK1, OTOG, DNM1
GO:0007596∼blood coagulation	4	0.045	5.0	SELP, ITGB3, GP1BA, F5
GO:1904153∼negative regulation of retrograde protein transport, ER to cytosol	2	0.047	40.5	UBE2G2, UBAC2
GO:0070862∼negative regulation of protein exit from endoplasmic reticulum	2	0.047	40.5	UBE2G2, UBAC2

**Table 3.** The table of GO of the genes encompassing KWB-specific nsSNPs. The main GO terms were DNA repair and cellular response to stress.

Term	Count	*P*-Value	Fold Enrichment	Genes
GO:0006974∼cellular response to DNA damage stimulus	29	2.69E-04	2.1	PIF1, DCLRE1C, PRKDC, BOD1L1, CIB1, XPC, ALKBH8, HELB, ASTE1, EME2, PMS1, FANCI, DTX3L, MGMT, SIRT4, RECQL, FOXN3, TNFRSF1B, PARP9, MLH3, WDR76, RINT1, RRM2B, TDP1, APLF, TIMELESS, MMS19, ATM, ERCC6
GO:0006281∼DNA repair	20	0.0014	2.2	FANCI, PIF1, DCLRE1C, DTX3L, PRKDC, MGMT, RECQL, XPC, PARP9, MLH3, HELB, RRM2B, ASTE1, EME2, TDP1, APLF, MMS19, ATM, ERCC6, PMS1
GO:0044773∼mitotic DNA damage checkpoint	6	0.0021	6.4	FANCI, RINT1, EME2, PRKDC, XPC, FOXN3
GO:0033554∼cellular response to stress	47	0.0023	1.6	SLC35B3, PIF1, DCLRE1C, PRKDC, HM13, BOD1L1, MRPS10, CIB1, XPC, FZD10, ALKBH8, TXN2, HELB, CREB3L3, OPA1, ASTE1, EME2, BOC, DVL2, UBXN8, PMS1, SASH1, GCN1, ZNF189, FANCI, DTX3L, MGMT, SIRT4, RECQL, EIF2AK3, FOXN3, TNFRSF1B, PARP9, MLH3, WDR76, GFAP, RINT1, TBL2, RRM2B, TDP1, APLF, SLCO1A2, NF1, TIMELESS, MMS19, ATM, ERCC6
GO:1904894∼positive regulation of STAT cascade	7	0.0027	4.9	IL22, CD40, CSF2, PECAM1, IL12B, IL19, JAK2
GO:0046427∼positive regulation of JAK-STAT cascade	7	0.0027	4.9	IL22, CD40, CSF2, PECAM1, IL12B, IL19, JAK2
GO:0044774∼mitotic DNA integrity checkpoint	6	0.0033	5.8	FANCI, RINT1, EME2, PRKDC, XPC, FOXN3
GO:0050790∼regulation of catalytic activity	43	0.0038	1.6	CYFIP2, ITIH4, PIF1, CD40, ECM1, ITIH2, LRRC19, LRTM1, PLEK, GDI2, CIB1, FZD10, SERPINA6, IQGAP2, ITIH1, IFIT1, ANTXR1, PKD1, RELN, RFK, DVL2, TBC1D10C, IL12B, JAK2, SASH1, GCN1, CAP2, TBC1D10B, SEMA4D, APAF1, MGMT, SIRT4, WEE2, SLPI, RTN4RL1, CCPG1, PPP1R1B, NF1, CTNNB1, LCP2, PKP4, ERCC6, PLIN5

## Discussion

Native pigs, including JNP, KNP, and KWB, are renowned for their good meat quality and juiciness. The fatty acid composition and higher redness value (CIE a*) explain the uniqueness of native pigs compared to other commercial pig breeds (Kim et al. 2009). In JNP, ion homeostasis, neuronal genes, and in KNP, cell adhesion-related genes can affect juicy and chewy pork (Sodhi et al. 2014, Kim et al. 2015).

Some native pigs are likely to be mixed with western commercial pig breeds, as mentioned above. In our study, some JNPs were mixed with DUR, which could interfere with the determination of breed-specific SNPs. Therefore, we eliminated some JNP individuals from further analysis. We did not find any compounded individuals in the NJ tree and treemix after elimination of some JNPs (Figure 1D and E).

Many SNPs were classified as breed-specific SNPs in the whole-genome sequencing data (Figure 2B and C). In the PCA using only breed-specific SNPs in JNP, KNP, and KWB, the results indicated obvious distinctness from other breeds, as seen in principal component 1 (Figure 3A, B, and C), implying that breed-specific SNPs can be useful genetic markers for genomics.

Breed-specific SNPs can elucidate the genomic landscape related to the breed’s unique characteristics in comparison to other breeds, which implies breed informative SNPs. Therefore, breed-specific SNPs may be useful genetic markers. Furthermore, because breed-specific nsSNPs alter amino acid sequence and protein function, they directly affect breed characteristics. The deleterious nsSNPs in the breed can actually be deleterious, but we identified that nsSNPs with tolerated effects can be the determining factors of the unique characteristics of native pig breeds.

To determine the genomic factors that explain the characteristics of native pig breeds in Korea, we surveyed the GO terms of each native pig breed (JNP, KNP, and KWB). In JNP, the notable genes containing nsSNPs were Transforming growth factor beta 2 proprotein (TGFB2), glutathione S-transferase kappa 1 (GSTK1) and ATP-binding cassette subfamily A member 8 (ABCA8). In previous study, TGFB2 gene were reported to be related to the developmental growth in JNP in comparison to the Berkshire pigs (Lee, Shin, et al. 2020). Members of the glutathione S-transferase (GST) family constitute a large class of cytoplasmic, membrane-bound, multigene, and multifunctional enzymes (Hayes et al. 2005). Sperm GSTs are not only important for sperm function but are also used as biomarkers for estimating sperm quality. The protein encoding GST kappa 1 (GSTK1, also named Dsb-L; nsSNP rs326967994: T→C, Ile→Val) is a highly conserved mitochondrial enzyme involved in lipid metabolism and energy production. Recent studies have suggested that GSTK1 plays an essential role in sperm function (Gao et al. 2009, Petit et al. 2013). ATP binding cassette subfamily A member 8 (ABCA8; nsSNP rs328587109: G→T, Ala→Ser) is a member of the ATP-binding cassette (ABC) transporter superfamily. ABCA8 is a transmembrane transporter responsible for the transport of organics such as drug efflux and cholesterol. This transcript is mediated by microRNA-374b-5p (miR-374b-5p). According to a luciferase reporter assay, miR-374b-5p binds to the ABCA8 3'-untranslated region (3’UTR) (Cui et al. 2020). The notable genes in breed-specific SNPs in JNP were calcium-dependent protein kinase II delta (CAMK2D: GO:0050801, ion homeostasis) and estrogen receptor 1 (ESR1: GO:0050801, ion homeostasis). CAMK2D regulates multiple ion channels by phosphorylation, including Ca2 + channels, sarcolemmal cardiac Na+ channels, and K+ channels. By activating these channels, CAMK2D causes an extracellular influx of ions (Grootjans et al. 2017). In a Chinese-European pig line, ESR1 was found to be related to litter size (Muñoz et al. 2007). ESR1 encodes an estrogen-and a ligand-activated transcription factor. The encoded protein regulates the transcription of many estrogen-inducible genes that play roles in growth, metabolism, gestation, and sexual development.

In KNP, the notable gene encompassing nsSNPs was integrin beta 3 (ITGB3, GO:0007155∼cell adhesion). Researchers have found that integrin beta 3 (ITGB3; nsSNP: rs341036511 A→G, Ile→Thr, rs336055648 T→C, Met→Val, rs339763435 T→ C, Tyr→Cys) affects immune cell adhesion and leukocyte movement (Jana et al. 2021). Integrins are ubiquitously expressed adhesion molecules that are highly glycosylated and contain Ca2 + or Mg2 + ions that are essential for ligand binding. Immunoglobulin superfamily containing leucine-rich repeat (ISLR) mRNA levels correlate negatively with LDL-cholesterol (Jacobsen et al. 2019). In KWB, the notable genes were DNA cross-link repair 1C (DCLRE1C, GO:0006974∼cellular response to DNA damage stimulus) and Deltex 3-like protein (DTX3L, GO:0006974∼cellular response to DNA damage stimulus). Severe combined immunodeficiency (SCID) is a group of inherited disorders characterized by T lymphocyte differentiation related to abnormal lymphocyte development. SCID caused by mutations in DCLRE1C (nsSNP: rs323944749 T→C, Tyr→His) results in the absence of both B and T cells (Iqbal et al. 2019). Previous studies have shown that DTX3L (nsSNP rs331172640 A → C, Leu→Arg) is more highly expressed in male pigs than in female pigs. DTX3L is a crucial member of the Notch signaling pathway that controls myogenesis (Zhang et al. 2013).

## Conclusion

Native pig breeds in Korea (Jeju Native Pig: JNP, Korean Native Pig: KNP, Korean Wild Boar: KWB) have unique characteristics, such as juicy and very tasty meat quality and disease resistance. We identified genes that represent the genomic characteristics of pigs native to Korea. In JNP, the notable GO term was neuron projection development, cell surface receptor signaling pathway, and ion homeostasis; in KNP, cell adhesion and wound healing; and in KWB, DNA repair and reproduction. Genes containing breed-specific SNPs may help explain the unique characteristics of native pig breeds.

## Availability of data and materials

The datasets analyzed during the current study are not publicly available because of intellectual property considerations, but are available from the corresponding author upon reasonable request.
